# Whole Genome Sequencing Analysis of *Bacillus thuringiensis* GR007 Reveals Multiple Pesticidal Protein Genes

**DOI:** 10.3389/fmicb.2021.758314

**Published:** 2021-11-02

**Authors:** Sabino Pacheco, Isabel Gómez, Marcos Chiñas, Jorge Sánchez, Mario Soberón, Alejandra Bravo

**Affiliations:** Departamento de Microbiología Molecular, Instituto de Biotecnología, Universidad Nacional Autónoma de México, Cuernavaca, Mexico

**Keywords:** genome sequencing, *Bacillus thuringiensis*, Cry toxin, MS/MS, biocontrol

## Abstract

*Bacillus thuringiensis* (*Bt*) are soil ubiquitous bacteria. They produce a great variability of insecticidal proteins, where certain of these toxins are used worldwide for pest control. Through their adaptation to diverse ecosystems, certain *Bt* strains have acquired genetic mobile elements by horizontal transfer, harboring genes that encode for different virulent factors and pesticidal proteins (PP). Genomic characterization of *Bt* strains provides a valuable source of PP with potential biotechnological applications for pest control. In this work, we have sequenced the complete genome of the bacterium *Bt* GR007 strain that is toxic to *Spodoptera frugiperda* and *Manduca sexta* larvae. Four replicons (one circular chromosome and three megaplasmids) were identified. The two largest megaplasmids (pGR340 and pGR157) contain multiple genes that codify for pesticidal proteins: 10 *cry* genes (*cry1Ab*, *cry1Bb*, *cry1Da*, *cry1Fb*, *cry1Hb*, *cry1Id*, *cry1Ja*, *cry1Ka*, *cry1Nb*, and *cry2Ad*), two *vip* genes (*vip3Af* and *vip3Ag*), two binary toxin genes (*vpa2Ac and vpb1Ca*), five genes that codify for insecticidal toxin components (Tc’s), and a truncated *cry1Bd*-like gene. In addition, genes that codify for several virulent factors were also found in this strain. Proteomic analysis of the parasporal crystals of GR007 revealed that they are composed of eight Cry proteins. Further cloning of these genes for their individual expression in *Bt* acrystalliferous strain, by means of their own intrinsic promoter showed expression of seven Cry proteins. These proteins display differential toxicity against *M. sexta* and *S. frugiperda* larvae, where Cry1Bb showed to be the most active protein against *S. frugiperda* larvae and Cry1Ka the most active protein against *M. sexta* larvae.

## Introduction

The *Bacillus cereus* sensu *lato* group encompasses several ubiquitous, endospore-forming Gram-positive bacteria including *B. cereus*, *B. anthracis*, and *Bacillus thuringiensis* (*Bt*). Despite their close phylogenetic relationship, their pathogenic properties are contrasting, since *B. anthracis* causes anthrax disease, *B. cereus* produces food poisoning in mammals, and *Bt* is a well-known pathogen of invertebrates (Helgason et al., 2000). Phylogenetic analysis showed that among the species of *B. cereus* sensu *lato* group, *B. cereus* and *Bt* are the closest bacteria. The main differences between them are found in their mobile genetic elements (MGE), such as insertion sequences, prophages, and plasmids. It was proposed that the acquisition of such MGE in *B. cereus* and *Bt* was facilitated by the absence of a functional CRISPR-Cas systems in many of the *B. cereus* sensu *lato* strains, allowing incorporation of novel genetic elements that may help for their better adaptation to diverse ecological niches (Zheng et al., 2020).

Due to their insecticidal properties, *Bt* have been exploited worldwide as biological pesticides for the control of multiple insect pests (Sanahuja et al., 2011). These bacteria are characterized by the production of pesticidal proteins (PPs) during their sporulation phase of growth. Most of these PPs accumulate as parasporal inclusions within the mother cell compartment and have been recently renamed in the Bacterial Pesticidal Protein Resource Center (BPPRC^1^) as follows: PPs belonging to the three domain crystal proteins retained the mnemonic name of Cry, while proteins related to Cyt family retained the mnemonic name of Cyt. PPs with homology to Etx-Mtx2 family are now named Mpp. The PPs with homology to aegerolysin are Gpp. Those with homology to the Toxin10/Bin family received the name of Tpp and the PPs with a predominantly alpha helical structure are named App (Crickmore et al., 2020). In addition, some *Bt* bacteria also produce and secrete additional PPs during their vegetative growth, such as proteins related to the vegetative insecticidal protein Vip3 that retained the mnemonic name of Vip, while the proteins related to the catalytic component of Vip2 are now named Vpa, and the PPs related to the Vip1 binding partner from the binary toxin are now recognized as Vpb (Crickmore et al., 2020). All these proteins are encoded in large megaplasmids as individual genes or grouped in pathogenic islands (PAI), accompanied by repeat sequences, insertion elements, and transposases, which may allow a higher recombination rate among diverse *Bt* strains (Mahillon and Chandler, 1998; Fiedoruk et al., 2017).

In searching novel PPs produced by *Bt*, that could be useful for pest control in agriculture or farming activities, a large number of *Bt* strains have been isolated from insect corpses, soil, and phylloplane. Characterization of such strains is not an easy task. Probably, the faster and low-cost approach to screen insecticidal toxins from a *Bt* strain collection is the traditional PCR strategy, either using a set of universal primers or designed specific primers (Cerón et al., 1994; Cerón et al., 1995; Porcar and Juárez-Pérez, 2003). In addition, also DNA-hybridization and DNA-microarray techniques were shown to be useful for the identification of specific *cry* genes (Beard et al., 2001; Letowski et al., 2005). However, these DNA-based techniques are limited to detect genes that were previously identified and lack information regarding to their expression. Proteomic analysis of parasporal crystal is more accurate to determine the presence and abundance of PPs expressed in specific *Bt* crystal inclusions, and such studies have been performed to identify the PPs produced by *Bt* subsp *galleriae* VKPMB-1757 and *wuhanensis* VKPMB-1226 strains (Chestukhina et al., 1994). Recently the advances in sequence strategies have shown that compete genome sequencing is the most efficient strategy to characterize interesting *Bt* strains and to discover novel PPs. Multiple *Bt* strains have already been characterized by using Illumina or PacBio strategies in order to obtain their complete genome sequence (Doggett et al., 2013; Liu et al., 2013; Palma et al., 2014; Gao et al., 2015; Cao et al., 2018).

*Spodoptera frugiperda* is a maize and rice insect pest that has become important worldwide, since this pest has recently migrated from America to Africa and Asia (Sun et al., 2021). This insect shows low susceptibility to Cry1Ac toxin from *Bt* but it is efficiently controlled with Cry1Fa toxin (Blanco et al., 2010). However, some populations of *S. frugiperda* have already evolved resistance to Cry1Fa-maize in different countries (Storer et al., 2012; Banerjee et al., 2017; Boaventura et al., 2020). Thus, the identification of additional toxins with high toxicity and no cross-resistance to Cry1Fa is needed to provide effective alternative tools for efficient control of this insect pest. *Bt* strain GR007 is a strain from our *Bt* strain collection toxic for lepidopteran insects including *S. frugiperda*. However, the identity of the PP in GR007 remains unknown. In this work, we have combined third-generation sequencing to obtain the complete genome of GR007 strain and used MS/MS proteomic analysis of its parasporal crystal characterization, in order to identify the expressed proteins. The *Bt* GR007 genome consists of four replicons: a circular chromosome and three extrachromosomal megaplasmids. Our analysis showed that the two large plasmids harbor multiple PPs arranged in PAIs. We have identified 20 genes encoding for PPs: 10 Cry proteins, two Vip proteins, two proteins that act as binary toxins (Vpa-Vpb), one Mpp protein and a cluster of five genes for insecticidal toxin components (Tc’s) that were originally described in *Photorhabdus luminescens* (Waterfield et al., 2001).

According to our proteomic analysis, the crystal inclusion of this strain is confirmed by eight Cry proteins. The genes for these Cry proteins were cloned to analyze their individual expression in *Bt* cells. Only seven Cry proteins were able to form parasporal crystals when expressed in *Bt* and all of them displayed insecticidal activity against to at least one lepidopteran species. Our work shows that combining these two approaches, whole genome sequence and LC-MS proteomic analysis of crystal inclusions, allows a complete characterization of novel *Bt* strains containing PPs with potential biotechnological applications.

## Materials and Methods

### Genomic DNA Purification

*Bt* GR007 strain was obtained from a recent screening of soil samples from the state of Morelos, Mexico, and selected for its toxicity against *S. frugiperda* larvae. *Bt* GR007 was cultured in LB liquid medium for 12 h at 30°C and bacteria were harvested by centrifugation. Genomic DNA was purified using AxyPrep Bacterial genomic DNA miniprep kit from Axygen (Corning Life Sciences, Glendale AZ) following the manufacturer’s instructions. DNA was analyzed by electrophoresis in agarose gel and quantified in NanoDrop 2000 spectrophotometer (Thermo Scientific). The 20 kb templates used for sequencing were prepared by using Blue Pippin Size-Selection System (Sage Science, Beverly, MA) that allows resolving and collecting high molecular weight DNA, according to the manufacturer’s instructions.

### DNA Sequencing and Assembly

The genomic sequence of *Bt* GR007 was obtained by Beijing Sinobiocore Biological Technology Co., using PacBio RSII platform. A total of 138,274 reads were obtained which represent 928,053,411 bp and total 151X fold coverage of the genome. All reads were assembled by using the Single Molecule, Real-Time (SMRT) sequencing data SMRTPipe v 2.3.0 from PacBio.^2^ Annotation of coding sequences (CDS), ribosomal RNA (rRNA), transfer RNA (tRNA), and miscellaneous RNA (miscRNA) were performed using Prokka v1.11 software (Seemann, 2014). The identity of CDS was searched using SwissProt and BLAST (nr/nt) databases. Classification of protein in Clusters of Orthologous Groups (COG) was performed using eggNOG-mapper (Huerta-Cepas et al., 2017). Insertion sequences were identified using ISFinder database (Siguier et al., 2006). Identification of ProPhages regions was performed using Phage_Finder (Fouts, 2006). Identification of CRISPR arrays were predicted using PILERCR v1.06 software (Edgar, 2007). Circular genome maps and plasmid comparison were generated using GView server (Petkau et al., 2010).

### Construction of Single Nucleotide Polymorphism Phylogenomic Tree

In this work we selected to work with the reported assembled genomes from different *Bt* strains that were found in the NCBI database. In this database we found genomic information of 670 Bt strains. However, only 63 genomes and 21 chromosomes are completely assembled. After taking out sequences that were duplicated, we downloaded 73 completely assembled genomes from this database. We used the Parsnp tool from the Harvest Suite software for fast multiple alignment of genomic sequences based on single nucleotide polymorphism (SNP) (Treangen et al., 2014). We ran Parsnp version 1.2 with the -c parameter. The phylogenomic tree was visualized and stylized in iTOL server (Letunic and Bork, 2019).

### Cloning of *Cry* Toxins

With the aim to clone the regulatory and terminator regions of each *cry* gene, PCR oligonucleotides were designed to amplify a fragment containing the *cry* gene flanked by ∼450 bp at 5′ and 3′ ends (Table 1). All PCR were performed with Phusion HD DNA Polymerase (Thermo Scientific Fisher) using as template the genomic DNA from *Bt* GR007 strain. The *cry* genes were cloned into pHT315 shuttle vector (Arantes and Lereclus, 1991) using In-Phusion HD Cloning Kit (Takara, Shiga, Japan) following the manufacturer’s instruction. Briefly, pHT315 linearized with *Sma*I endonuclease was fused with the PCR fragments using In-Phusion Kit enzyme premix during 15 min at 50°C, and the cloning reaction was electrotransformed into *E. coli* DH5α cells. Transformant cells were analyzed by colony PCR and plasmids of positive clones were purified and further sequenced.

**TABLE 1:** List of oligonucleotides for cloning the *cry* genes.

**Primer**	**Sequence (5′→3′)**
1AbF	CTCTAGAGGATCCCCTCGAAAATCTTTTTGCTATCTATGG
1AbR	TCGAGCTCGGTACCCTTCAAGATGAATTGCAGGTAAATGG
1BbF	CTCTAGAGGATCCCCCAAAAAGGCACCTTATGTTTTGTG
1BbR	TCGAGCTCGGTACCCTAATATATGTAAAACAGGAAGAATG
1DaF	CTCTAGAGGATCCCCCTGTATATCTAACAATTAAGATG
1DaR/FbR	TCGAGCTCGGTACCCGAATGACATAAATTTTTGTCATCGC
1FbF	CTCTAGAGGATCCCCGATGAATTACAGATAAATGGTCTAC
1HbF	CTCTAGAGGATCCCCTAAACCTACATAAATGTGACTGTC
1HbR	TCGAGCTCGGTACCCTTATTCTTCTATATCTATAAAATC
1IdF	CTCTAGAGGATCCCCATCTAAGCCAGTAATAGGGC
1IdR	TCGAGCTCGGTACCCGAAAAATGGACATTTTCCTCC
1JaR	TCGAGCTCGGTACCCCTTTATGTTGTATATTTTTCGAACGG
1JaF	CTCTAGAGGATCCCCTTTTAGATGAATTACAAGTAAATGG
1KaF	CTCTAGAGGATCCCCTCAATGCATACTTTCAATAAGGAG
1KaR	TCGAGCTCGGTACCCTTATGAGCTGCTATCAAAAGG
1NbF	CTCTAGAGGATCCCCAATTGGAGTACAGACGAGAG
1NbR	TCGAGCTCGGTACCCGAACAACGTCAATTTGAAGGG
2AdF	CTCTAGAGGATCCCCGTCTCAGGCATAAGTAATGAGG
2AdR	TCGAGCTCGGTACCCGTGTTTTGATTGGTGTCCCG

### Parasporal Crystal Production

Plasmids harboring *cry* genes were electrotransformed into the acrystalliferous *Bt* strain 407, as previously described (Macaluso and Mettus, 1991). *Bt* strains were grown during 72 h at 30°C in HCT medium (Lecadet et al., 1980) supplemented with 10 μg/ml erythromycin. After sporulation, spore/crystal mixture was recovered and washed three times with wash solution (300 mM NaCl, 10 mM EDTA) and three times with 1 mM PMSF. After these washing steps the spore/crystal mixture was then suspended in ddH_2_0. These samples were directly suspended in SDS-PAGE Laemmli buffer, heated 3 min at 100°C, and analyzed by 10% SDS-PAGE. Protein concentration of the final spore/crystal mixtures suspended in ddH_2_0 was estimated by Bradford method using a BSA standard curve as reference.

### Liquid Chromatography-Mass Spectrometry Analysis

Spore/crystal inclusion suspensions of *Bt* GR007 were mixed in Laemmli loading buffer, boiled for three min, and proteins were separated on a 10% SDS-PAGE. Protein bands were excised, digested “in gel” with trypsin, and desalted. Cleaved peptides were subsequently analyzed by Liquid Chromatography-Mass Spectrometry (LC-MS) system composed of a nanoflux pump EASY-nLC II and mass spectrophotometer LTQ-Orbitrap Velos (Thermo Fisher) at the proteomic facility of the Institute of Biotechnology from National Autonomous University of Mexico. For protein identification, data were screened against the Cry and Vip protein sequences obtained from genome sequencing of this work using the Proteome Discoverer software. A minimum False Discovery Rate (FDR) of 0.01 y maximum FDR of 0.05 were used for peptide identification.

### Bioassays

*Manduca sexta* and *Spodoptera frugiperda* insect colonies were reared on artificial diets (Bell and Joachim, 1976; Villegas-Mendoza and Rosas-García, 2013), under controlled conditions of relative humidity (70–80%), temperature (25 ± 2°C) and photoperiod (10 h/14 h, light/dark). Bioassays were performed with neonate larvae using six different doses of the spore/crystal mixture that were poured on the surface of the diet. For bioassays against *M. sexta* larvae we used the following concentrations: 70, 35, 17.5, 8.7, 4.3, and 2.1 ng/cm^2^. For bioassays against *S. frugiperda* larvae we used the following concentrations: 2000, 1000, 500, 250, 125, and 62 ng/cm^2^. The Cry1Ka y Cry1Hb proteins were tested up to 5000 ng/cm^2^. The negative control was water added to the surface of the diet. We used 24 well polystyrene plates and one plate was used per each concentration of spore/crystal dose in triplicate (a total of 72 larvae were used per toxin concentration in each repetition, thus in each bioassay 432 larvae were analyzed for the 6 different concentrations of spore/crystal mixtures and 72 larvae for the negative control). Mortality was analyzed after 7 days and the 50% lethal concentration (*LC*_50_) was calculated with Probit LeOra software. Mortality of the control was lower than 5%. Complete bioassays with three technical replicates were performed in duplicate (864 larvae in total per bioassay performed with each toxin).

## Results

### Genome and Phylogenetic Analysis of *Bt* GR007

**FIGURE 1:**
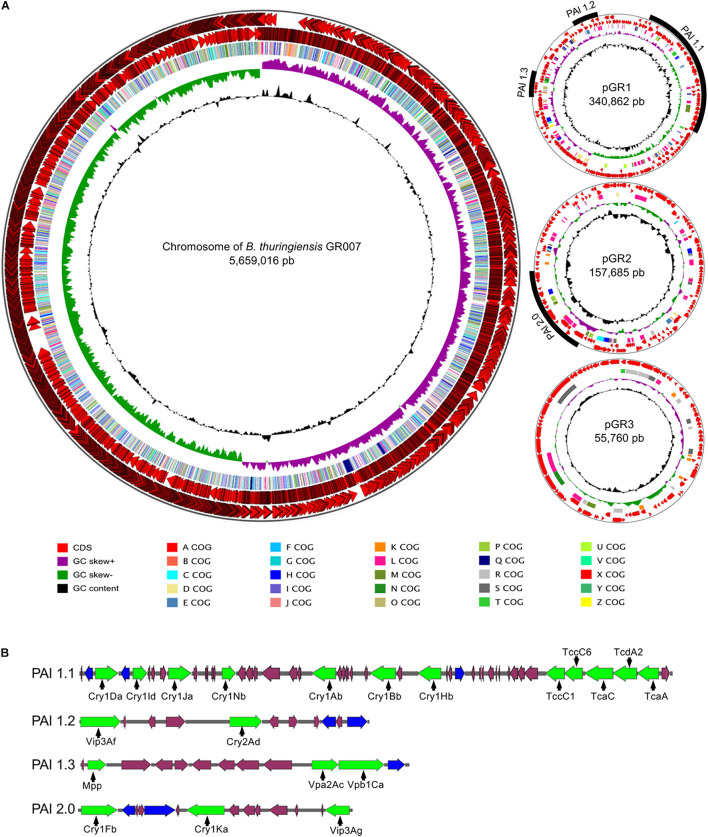
Genome of *B. thuringiensis* strain GR007. **(A)** Circular maps of chromosome and plasmids. The distribution of circles from outside to inside indicate CDS forward, CDS reverse, COG functional assignation, GC skew, GC content. The pathogenic islands (PAI) identified are indicated in the plasmids pGR340 and pGR157. **(B)** Representation of PAIs. Pesticidal proteins are represented in green arrows, transposases in blue arrows and additional ORF into the PAIs with purple arrows.

*Bt* GR007 strain was selected due to its toxicity against *S. frugiperda* during preliminary screening assays compared to other native strains. In order to gain access to the gene sequences of potential PP in *Bt* GR007 strain, the whole genome of this strain was obtained and assembled (BioProject: PRJNA736034). The genome of this strain consists of four replicons, the chromosome and three plasmids (Figure 1A). The circular chromosome contains 5,659,016 bp with 36.2% GC content (Gene Bank Accession number CP076539). A total number of 5,714 CDS were predicted, from which 4,746 proteins were identified using the database of SwissProt and BLAST analysis. The predicted proteins were further classified with COG functional assignation using eggNOG database. The chromosome also contains 30 rRNAs (10 23S, 10 16S, and 10 5S), 104 tRNAs, 145 miscRNAs, and one tmRNA. DNA regions related with genetic mobility were found along the chromosome, among these we found seven large prophage regions and 89 insertion sequences for transposases (Tnp). In agreement with most of the *Bt* strains that have been sequenced previously, *Bt* GR007 lacks a functional CRISPR system (Zheng et al., 2020; Table 2).

**TABLE 2:** Features of the genome of *Bacillus thuringiensis* GR007 strain.

	**Chromosome**	**pGR340**	**pGR157**	**pGR55**
Size (bp)	5,659,016	340,862	157,685	55,760
GC content (%)	36.2	34.5	33.5	35.5
CDS	5,714	290	154	77
rRNA	30	–	–	–
tRNA	104	–	–	–
miscRNA	145	8	1	–
tmRNA	1	–	–	–
COG assignation	4,746	150	75	25
Pro-phages	7	–	–	–
Insertion sequences	89	19	9	–
CRISPR arrays	–	–	–	–

The three extrachromosomal plasmids pGR340, pGR157, and pGR55 consist of 340,862, 157,685, and 55,760 bp, respectively (Figure 1A) (Gene Bank accession numbers CP076541, CP076540, and CP076542). The two largest plasmids, pGR340 and pGR157, harbor the genes that encode for potential PPs, which are grouped in PAIs, and their annotation was done according to the new database of PPs (see text footnote 1). The plasmid pGR340 contains three PAIs designed as PAI-1.1, PAI-1.2, and PAI-1.3 (Figure 1B). The length of PAI-1.1 is 88.2 kbp and contains seven *cry* genes encoding for lepidopteran specific Cry proteins (Cry1Da, Cry1Id, Cry1Ja, Cry1Nb, Cry1Ab, Cry1Bb, and Cry1Hb) and a cluster of five genes codifying for insecticidal toxin components (Tcs) (TccC1, TccC6, TcaC, TcdA2, and TcaA). The PAI-1.2 of 17.3 kbp contains two genes encoding for PPs, named Vip3Af and Cry2Ad. The PAI-1.3 with a length of 16.5 kbp has an homologous gene that codifies for a protein related to the Etx/Mtx2 toxin family (recently designed as Mpp). This PAI-1.3 island also contains genes that codify for the binary toxins Vpa2Ac and Vpb1Ca (old names were Vip2Ac and Vip1Ca, respectively) arranged in an operon. The plasmid pGR157 contains a single PAI named PAI2.0 of 26.5 kbp that contains two genes encoding for Cry proteins (Cry1Fb and Cry1Ka) and one gene encoding for a Vip protein (Vip3Ag). In addition, pGR157 contains a gene for a non-functional truncated Cry1Bd-like protein since it lacks the N-terminal region that is present in the Cry1Bd protein (NCBI Acc. No AAD10292). All PAIs were associated with Tnp’s (displayed as blue arrows in Figure 1B). Supplementary File 1 contains the detailed information of *Bt* GR007 genome annotation.

*Bt* encodes a large number of virulence-associated genes. We searched for these genes and found that most of them were found in the chromosome of GR007 strain (Table 3), while some virulence factors were found in PAI 1.1 such as cytolysin operon (*hblL2*, *hblL1*, and *hblB*), the phospholipase C (*plc-6*), subtilisin (*sub-8*), the cell wall binding/hydrolysis protein (*scwl-9*), and internalin (*inlA-5*) (Table 3). The PAI2.0 island also codifies for a cell wall hydrolase (*cwlJ-3*) (Table 3).

**TABLE 3:** Genes coding for virulence factors in *Bacillus thuringiensis* GR007 genome.

**Virulence factor**	**Location**	**Start**	**Stop**	**Strand**
**Insecticidal toxins**				
*cry1Ab*	pGR340	56239	59772	−
*cry1Bb*	pGR340	65045	68878	−
*cry1Da*	pGR340	23248	26742	+
*cry1Fb*	pGR340	91003	94509	+
*cry1Hb*	pGR340	72302	75811	−
*cry1Id*	pGR340	28968	31127	+
*cry1Ja*	pGR340	34394	37897	+
*cry1Nb*	pGR340	42574	44589	+
*cry1Ka*	pGR157	101159	104803	−
*cry2Ad*	pGR340	321333	323234	+
Operon: *vpa2Ac* and *vpb1Ca*	pGR340	266106	269799	+
*vip3Af*	pGR340	312503	314863	+
*vip3Ag*	pGR157	114534	116891	−
Operon Tc toxin: *tccC1*, *tccC6*, *tcaC*, *tcdA2* and *tcaA*	pGR340	91811	108969	−
*mpp* (Etx/Mtx2 toxin)	pGR340	254591	255532	+
**Cytolysins**				
Operon *hblL1*, *hblL2* and *hblB*	Chromosome	1783884	1787477	+
Operon *hblB-1*, *hblB-2*, *hblL1* and *hblL2*	Chromosome	3194811	3200351	−
Operon *hblL2*, *hblL1* and *hblB*	pGR340	6505	10280	+
*cylI*	Chromosome	5145470	5146522	−
*hblL2*	Chromosome	2278822	2279034	+
*hly II*	Chromosome	3616020	3617258	−
*hly III-1*	Chromosome	2196784	2197440	−
*hly III-2*	Chromosome	2241080	2242435	+
*hly III-3*	Chromosome	5619318	5619965	−
*hly III-4*	Chromosome	607229	608527	+
*hly III-5*	Chromosome	1987084	1988391	−
*xhlA-1*	Chromosome	2046160	2046384	+
*xhlA-2*	Chromosome	3511827	3512051	−
*xhlA-3*	Chromosome	5038539	5038763	−
*xhlA-4*	Chromosome	2046160	2046384	+
*xhlA-5*	Chromosome	2662576	2662809	+
*tlyA*	Chromosome	4313857	4314696	−
*alo* (Alveolysin)	Chromosome	5219805	5221343	−
**Chitinases**				
*chiA*	Chromosome	423999	426023	−
*chiD*	Chromosome	3832278	3833360	+
**Chitin-binding proteins**				
*cbp-1*	Chromosome	2761440	2762807	+
*cbp-2*	Chromosome	2481050	2483011	+
*cbp-3*	Chromosome	2924336	2925001	−
*cbp-4*	Chromosome	3580901	3582277	−
**Phospholipases**				
*plc-1* (Phospholipase C)	Chromosome	675105	675956	+
*plc-2* (Zinc-dep. Phospholipase C)	Chromosome	2101245	2101883	−
*plc-3* (Phospholipase C)	Chromosome	3573651	3574487	+
*plc-4* (Phospholipase C)	Chromosome	3580098	3580628	+
*plc-5* (Phospholipase C)	Chromosome	3871108	3872088	−
*plc-6* (Phospholipase C)	pGR340	256339	257829	+
*spl* (Sphingomyelinase C)	Chromosome	676033	677034	+
*plA2-1* (Phospholipase A2)	Chromosome	752881	753735	+
*plA2-2* (Phospholipase A2)	Chromosome	1935987	1936952	−
*plA2-3* (Phospholipase A2)	Chromosome	2909139	2909990	+
*plbA* (Lysophospholipase L1)	Chromosome	3469581	3470147	−
*plbB-1* (Lysophospholipase L2)	Chromosome	1766382	1767305	+
*plbB-2* (Lysophospholipase L2)	Chromosome	4842218	4843021	−
*pld* (Phospholipase D)	Chromosome	1709427	1710638	+
**Metalloproteases**				
*inhA-1*	Chromosome	668528	670927	+
*inhA-2*	Chromosome	1272828	1275215	+
*inhA-3*	Chromosome	3078491	3080878	−
**Enhancins**				
*be1-1*	Chromosome	2481050	2483011	+
*be1-2*	Chromosome	3604691	3606898	−
*be1-3*	Chromosome	5188499	5192929	−
**Camelysins**				
*cotN*	Chromosome	1269383	1269970	+
*calY-1*	Chromosome	1271282	1271875	+
*calY-2*	Chromosome	2530523	2531122	+
*calY-3*	Chromosome	4977485	4977613	+
**Bacillolysins**				
*npr-1*	Chromosome	602815	604515	+
*npr-2*	Chromosome	2168785	2170296	+
*npr-3*	Chromosome	2576618	2579299	+
*npr-4*	Chromosome	2858944	2860647	+
*npr-5*	Chromosome	5167680	5169350	−
**Subtilisins**	Chromosome	5505788	5507539	+
*sub-1*	Chromosome	1054733	1055704	+
*sub-2*	Chromosome	1943955	1944902	+
*sub-3*	Chromosome	2320309	2321502	−
*sub-4*	Chromosome	2526021	2530262	+
*sub-5*	Chromosome	3467836	3469212	−
*sub-6*	Chromosome	3872268	3874109	−
*sub-7*	Chromosome	4463982	4466735	+
*sub-8*	pGR340	135923	137302	−
**Inhibitor of autoinducer**				
*aiiA*	Chromosome	3505497	3506249	−
**Collagenases**				
*colA-1*	Chromosome	544515	547412	+
*colA-2*	Chromosome	3467836	3469212	−
*colA-3*	Chromosome	3628281	3631196	−
*colA-4*	Chromosome	3872268	3874109	−
*colA-5*	Chromosome	4488977	4490257	−
*colA-6*	Chromosome	4490276	4491205	−
**Collagen-binding proteins**				
*scol-1*	Chromosome	1075550	1082065	+
*scol-2*	Chromosome	2584166	2586403	+
*scol-3*	Chromosome	3620861	3624142	−
*scol-4*	Chromosome	5193488	5194411	−
*scol-5*	Chromosome	5512675	5522325	+
**Cell-wall hydrolases**				
*cwlA-1*	Chromosome	1900524	1901315	+
*cwlA-2/ampD*	Chromosome	2919865	2920695	−
*cwlA-3/ampA*	Chromosome	347826	348644	+
*cwlA-4/xlyB*	Chromosome	5037337	5038038	−
*cwlB-1*	Chromosome	1466331	1466861	+
*cwlB-2*	Chromosome	2921124	2921858	−
*CwlC*	Chromosome	2205925	2206911	+
*CwlD*	Chromosome	146155	146868	+
*CwlH*	Chromosome	879264	880967	−
*cwlJ-1*	Chromosome	2616182	2616610	−
*cwlJ-2*	Chromosome	5551941	5552363	+
*cwlJ-3*	pGR157	79763	80119	+
*cwlK/lysA*	Chromosome	2723888	2724733	+
*cwlO-1*	Chromosome	1407593	1408867	−
*cwlO-2*	Chromosome	5337751	5339178	−
*cwlS-1*	Chromosome	1858460	1859752	+
*cwlS-2*	Chromosome	5382632	5384374	+
**Cell-wall binding/hydrolysis proteins**				
*scwl-1*	Chromosome	468610	469503	+
*scwl-2*	Chromosome	890854	892716	+
*scwl-3*	Chromosome	897724	899331	+
*scwl-4*	Chromosome	1001965	1003698	+
*scwl-5*	Chromosome	1938305	1939465	+
*scwl-6*	Chromosome	2515126	2516544	+
*scwl-7*	Chromosome	3617563	3618795	−
*scwl-8*	Chromosome	3904515	3906698	+
*scwl-9*	pGR340	90504	91151	−
**Internalins**				
*inlA-1*	Chromosome	539542	542529	+
*inlA-2*	Chromosome	1131972	1132628	+
*inlA-3*	Chromosome	1132870	1133532	+
*inlA-4*	Chromosome	1321347	1323629	+
*inlA-5*	pGR340	184518	185762	−

**FIGURE 2:**
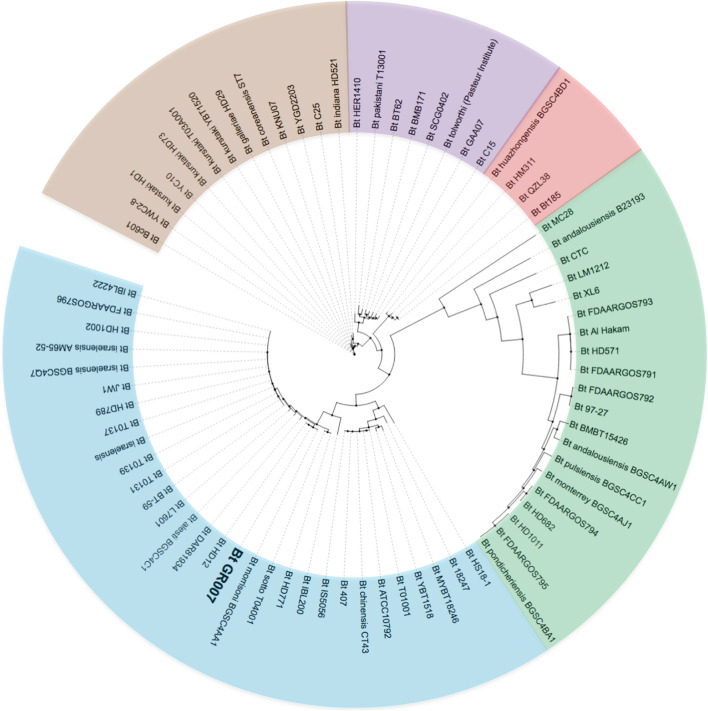
Unrooted phylogenomic tree based on SNP of core genomes of GR007 and other 73 *B. thuringiensis* strains from NCBI that were reported as assembled genomes. Clades are represented with blue, green, red, purple, and brown colors.

To investigate the phylogenetic relationship of *Bt* GR007 strain with other previously sequenced *Bt* strains, we constructed a phylogenomic tree based on SNP of their circular chromosomes (Figure 2). Figure 2 shows that *Bt* GR007 strain was grouped in a clade of *Bacillus* strains that kill insects and nematodes, such as *Bt* subsp. *israelensis* (Dipteran-specific), *Bt* subsp. *morrisoni* BGSC 4AA1 (Colepteran-specific), and *Bt* YBT-1518 (Nematoda-specific). However, the overall structure of this phylogenomic tree showed a low phylogenetic correlation among the linage of *Bt* GR007 with other well characterized Lepidopteran-specific *Bt* strains, such as the HD1, HD73, and YBT1520 strains that were grouped in a different branch. Based on the data of the phylogenomic tree, the closest *Bt* strain to GR007 was *Bt* HD12 strain. The Cry proteins content of *Bt* GR007 is similar to *Bt* HD12 (BioProject: PRJNA302106), with the exception that the latter strain lacks *cry1Fb* and *vip3Ag* genes. In addition, the number of plasmids is different between these two strains, since *Bt* HD12 contains six plasmids (named as pHD120017, pHD120038, pHD120039, pHD120112, pHD120161, and pHD120345) (Gene Bank Accession Numbers CP014848.1 to CP014853.1). A detailed analysis of the plasmids present in these two strains showed that pGR340 from GR007 strain was essentially identical to pHD120345 from HD12 strain (AN CP014853.1) and contains the same number of the PAIs, while pGR157 and pGR55 from GR007 strain matched with pHD120161 from HD12 strain (AN CP014852.1), suggesting a recombination of these two plasmids in *Bt* GR007 to form a single plasmid lacking *cry1Fb* and *vip3Ag* genes (pHD120161) in *Bt* HD12 strain (Supplementary Figure 1).

### Proteomic Analysis of the Proteins Expressed in the Crystal Inclusion of GR007

**FIGURE 3:**
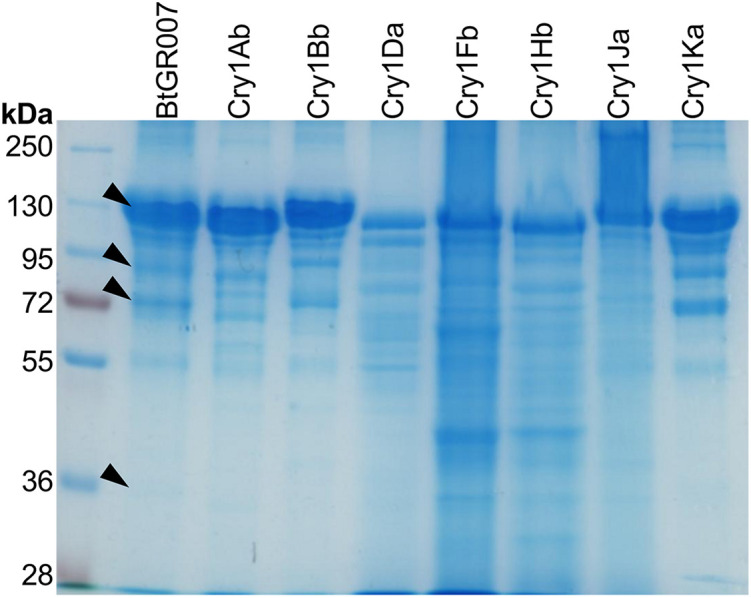
SDS-PAGE of parasporal crystal proteins. Proteins of *Bt* GR007 analyzed by LC-MS are labeled with black arrows. The *cry* genes codifying for Cry1Ab, Cry1Bb, Cry1Da, Cry1Fb, Cry1Hb, Cry1Ja, and Cry1Ka proteins were cloned in pHT315 shutter vector and electrotransformed into acrystalliferous *Bt* 407 strain. All transformant *Bt* strains were grown until sporulation and the spore/crystal mixture of these strains were analyzed by SDS-PAGE. Cry1Ab, Cry1Bb, Cry1Da, Cry1Fb, Cry1Hb, Cry1Ja, and Cry1Ka correspond to the protein profiles observed in each one of these *Bt* transformant strains.

A proteomic analysis was performed to analyze the PP’s composition of the parasporal crystal produced by *Bt* GR007 strain. The strain was grown until the sporulation phase and parasporal crystals were separated by SDS-PAGE. Proteins with apparent molecular weight of 130, 95, 72, and 32 kDa, which correspond to the molecular weight of PPs identified in the megaplasmids, were excised directly from the gel after Coomassie blue staining (Figure 3, lane *Bt* GR007) and analyzed by LC-MS analysis. Peptides identified by LC-MS matched to eight Cry proteins (Cry1Ab, Cry1Bb, Cry1Da, Cry1Fb, Cry1Hb, Cry1Ja, Cry1Ka, and Cry1Nb) showing high sequence coverage in the 130 and 72 kDa bands, that correspond to protoxin and activated toxin, respectively (Table 4).

**TABLE 4:** Proteomic analysis of *B. thuringiensi*s GR007 crystal composition.

		**% of sequence coverage in protein bands**			
	**MW (kDa)**	**130 kDa**	**95 kDa**	**72 kDa**	**32 kDa**
Cry1Ab	133.3	52	23	51	22
Cry1Bb	145.0	46	14	46	9.4
Cry1Da	131.9	46	19	43	10
Cry1Fb	132.3	46	20	46	15
Cry1Hb	132.5	24	9	17	4.6
Cry1Ja	132.6	50	26	50	16
Cry1Ka	137.1	63	36	49	21
Cry1Nb	76.9	6	4	17	4.8

### Characterization of Pesticidal Cry Proteins

To further analyze the Cry proteins expressed in *Bt* GR007, we cloned all *cry* genes identified in this strain to evaluate their toxicity when expressed in the acrystalliferous *Bt* strain 407. The *cry* genes codifying for Cry1Ab, Cry1Bb, Cry1Da, Cry1Fb, Cry1Hb, Cry1Ja, Cry1Ka, and Cry1Nb proteins were identified in the pGR340 and pGR157 megaplasmids. Oligonucleotides were designed to amplify *via* PCR the complete gene including a DNA fragment approximately 450 bp upstream of the start codon of the gene, and 450 bp after the stop codon. We expected that these DNA fragments should contain the intrinsic promotor and terminator regions from each *cry* gene for their individual expression. These DNA fragments were cloned in pHT315 shutter vector and electrotransformed into acrystalliferous *Bt* 407 strain. All transformant *Bt* strains were grown until sporulation and the spore/crystal mixtures were analyzed by SDS-PAGE. Cry1Ab, Cry1Bb, Cry1Da, Cry1Fb, Cry1Hb, Cry1Ja, and Cry1Ka proteins were successfully produced (Figure 3). The Cry1Id and Cry2Ad that were not identified in the parasporal crystal were also cloned, but these proteins were not found as inclusion bodies when transformed into Bt 407 (Supplementary Figure 2).

We analyzed the promotor region of these genes, the upstream nucleotide sequence was aligned with the *cry1Ab* promoter region that is depend in Sig^*K*^ or Sig^*E*^ factors for its expression. The promotor regions of *cry1Da, cry1Ja, cry1Bb, cry1Hb, cry1Fb*, and *cry1Ka* are similar to *cry1Ab*, while promotor regions from *cry1Nb, cry1Id*, and *cry2Ad* showed different sequences, suggesting that their regulation is not dependent on Sig^*E*^ and Sig^*K*^ factors (Supplementary Figure 3).

The toxicity of GR007 strain was tested against two lepidopteran insects, *S. frugiperda* an *M. sexta.* We also assayed insecticidal activity against the dipteran insect, *Aedes aegypti.* Toxicity of this strain was confirmed only for the two lepidopteran larvae while no toxicity was observed against dipteran insects (data not shown). Finally the toxicity of the individual Cry1Ab, Cry1Bb, Cry1Da, Cry1Fb, Cry1Hb, Cry1Ja, and Cry1Ka proteins was analyzed in bioassays against *S. frugiperda* an *M. sexta* larvae. Table 5 shows that all Cry toxins were highly toxic to *M. sexta* larvae but showed differential toxicity against *S. frugiperda*. The Cry1Bb protein was the most active toxin against *S. frugiperda* with an *LC*_50_ value of 128.5 ng/cm^2^ while Cry1Hb and Cry1Ka showed no toxicity even at the highest concentration of protein that was used in the bioassay (5000 ng/cm^2^) (Table 5).

**TABLE 5:** Toxicity of parasporal crystals against insect larvae.

**Protein**	***Manduca sexta***	***Spodoptera frugiperda***
***Bt* GR007**	11.6 (6.3–16.0)	1,200 (900–1,800)
Cry1Ab	8.6 (6.0–11.0)	344.2 (270.3–450.9)
Cry1Bb	5.4 (0.5–12.5)	128.5 (114.5–152.1)
Cry1Da	6.4 (4.9–8.0)	942.9 (694.0–1,783.4)
Cry1Fb	7.0 (5.6–8.7)	622.1 (405.0–456.3)
Cry1Hb	7.6 (5.4–9.7)	>5,000
Cry1Ja	5.5 (4.3–7.0)	1,005 (850.4–1,229.1)
Cry1Ka	2.1 (0.5–3.3)	>5,000

*Values correspond to *LC*_50_ in ng/cm^2^ and 95% fiducial limits are shown in parenthesis.*

## Discussion

Whole genome sequence screening of *Bt* strains has been one of the most successful approaches for PP gene discovery in recent years. In addition, it has provided data for the analysis of the evolution of the *Bt* genome and analysis of horizontal gene transfer events that have contributed to the evolution of *Bt* strains (Zheng et al., 2017). *Bt* bacteria are characterized by the presence of insecticidal proteins that accumulate in crystal inclusions during their sporulation phase of growth and such proteins are codified in different plasmids. It was shown that the *B. cereus* group selectively inactivated the CRISPR Cas system, which correlates with acquisition of mobile elements, such as different plasmids containing genetic information that could help in the adaptation of these bacteria to diverse environments (Zheng et al., 2020). *Bt* GR007 strain did not contain a functional CRISPR Cas system, which correlated with its high PP gene content, encoded in different plasmids. Phylogenetic analysis of whole genomes showed that *Bt* GR007 strain (BioProject: PRJNA736034) is closely related with *Bt* HD12 strain (BioProject: PRJNA302106). Interestingly, the genome sequence comparison between both strains suggested that HD12 might had a genome rearrangement where information contained in plasmids pGR157 and pGR55, that are present in *Bt* GR007 strain, recombined to form a single large plasmid named pHD12016 in HD12 strain, loosing *cry1Fb* and *vip3Ag* genes during this recombination process.

It is now recognized that different Bt strains harbor several PP that in theory may expand their host specificity by improving its toxic activity against different insect targets (Wang et al., 2020). *Bt* GR007 strain showed insecticidal activity against lepidopteran insects. This insect specificity correlated with the identification of Cry1 proteins expressed in the crystal inclusions that are specific against lepidopteran insects (Crickmore et al., 2020). However, other PP genes found in PAI-1.3 island of this strain, such as *vpa2Ac* and *vpb1Ca* genes and the putative novel *mpp* gene, suggest that it may also display also coleopteran toxicity, but this hypothesis remains to be studied in the future.

The genomic analysis allowed the identification of multiple PAI islands. The PAI-1.1 is the longest PAI region in this strain, containing seven different *cry* genes. All of them codify for lepidopteran specific Cry proteins (Cry1Da, Cry1Id, Cry1Ja, Cry1Nb, Cry1Ab, Cry1Bb, and Cry1Hb) (Crickmore et al., 2020). This PAI-1.1 also codifies for a cluster of five insecticidal toxin components (Tcs). The Tcs proteins were originally identified in enterobacteria *P. luminescens* and *Xenorhabdus nematophila*, which are symbiont of nematodes (Waterfield et al., 2001). These Tcs proteins are pore-forming toxins that kill lepidopteran and dipteran insects. It was previously shown that *tcs* genes may be found in other bacteria such as *Serratia entomophila* that is also an insect pathogen or *Yersinia pestis*, that is mammalian pathogen transmitted by an insect vector (Waterfield et al., 2001). It was also reported that some *Bt* strains may harbor these genes and that *tcaA* and *tcaB* genes were expressed during the infection of gypsy moth larvae by these *Bt* strains (Blackburn et al., 2011). In the case of GR007 strain, it still remains to be demonstrated if these Tcs proteins participate in toxicity.

The PAI-1.2 contains only two genes encoding for PPs (Vip3Af and Cry2Ad), which are also recognized for their toxicity against lepidopteran insects (Estruch et al., 1996; Liao et al., 2015). In contrast the PAI-1.3 codifies for proteins that have been associated with toxicity against different insect orders, like the PP that shows 34.6% identity with Mpp4Aa from *Lysinibacillus sphaericus* that belongs to the Mtx2-protein family active against mosquito larvae (Rey et al., 2016). In *Bt* the Mtx2 like-toxins (now named as Mpp or Gpp) are toxic to other insect orders, such as the Mpp64Ba and Mpp64Ca toxins (previously known as Cry64Ba and Cry64Ca) that are highly active against hemipteran pests (Liu et al., 2018), or Gpp34Aa/Tpp35Aa proteins (previously known as Cry34/Cry35) that showed toxicity against coleopteran pests (Kelker et al., 2014). However, the new PP found in GR007 strain was not classified in the BPPRC web site (see text footnote 1), since we lack information of its pesticidal activity and its identity with other *Bt* PPs is lower than 35%. This PAI-1.3 island also contains genes that codify for the binary Vpa2Ac and Vpb1Ca toxins, that were previously shown to be toxic to coleopteran larvae (Bi et al., 2015). However, all these PP proteins were not detected in the parasporal crystal inclusion of *Bt* GR007 so their expression and insect specificity remains to be analyzed. Finally The PAI2.0 island contains three genes encoding for PP that have been shown to be toxic to lepidopteran larvae (Cry1Fb, Cry1Ka, and Vip3Ag) (Koo et al., 1995; Estruch et al., 1996; DaSilva et al., 2016). The Vip3A proteins are expressed in the vegetative phase of growth and are secreted into the medium. The expression of Vip3Ag and Vip3Af in GR007 strain remains to be characterized.

All PAIs from GR007 strain showed multiple Tnp’s sequences (blue arrows in Figure 1B). It has been proposed that Tnp sequences have contributed to the transfer of PP genes and their recombination among differed *Bt* strains to generate the great variability of these proteins (Mahillon and Chandler, 1998).

Analysis of non-toxin virulence factors produced by *Bt* GR007 strain showed that most of them are codified in the chromosome of this bacterium (Table 3). It has been proposed that Chitinases, collagenases, cell wall hydrolases, phospholipases, and proteases such as metalloproteases, camelysin (a surface metalloproteinase), bacillolysins (a neutral proteinase), and subtilisin (a serine protease) may be important for efficient larval body utilization during the infection process (Malovichko et al., 2019). Enhancin and collagenase proteins may also participate in the utilization of insect tissues during the last stages of saprophytic colonization (Malovichko et al., 2019). The cytolysins such as *hblL2*, *hblL1*, and *hblB*, a three-component hemolytic complex, was shown to be produced by *B. cereus sensu lato* strains and has been implicated as a cause of diarrhea associated with food poisoning (Worthy et al., 2021). These cytolysin genes and other cytolysins such as *hly II and hly III, xhlA, tlyA* were found in the chromosome of GR007 strain. Finally, the internalin proteins were initially described in *Listeria monocytogenes*, playing an important role in cell invasion (Dramsi et al., 1997). In the case of *B. cereus* it was proposed that internalins may play a role during pathogenesis and that they are induced in insects after oral ingestion (Fedhila et al., 2006). All these virulence factors may potentially add competitive advantages to the *Bt* strains, improving their toxicity to the target insect as previously suggested (Malovichko et al., 2019).

Here, we cloned the *cry* genes found in GR007 strain and analyzed their expression and toxicity against two lepidopteran insects, *M. sexta* and *S. frugiperda*. The toxicity of the *Bt* GR007 strain to these insect pests was relatively low compared to the toxicity of some Cry proteins expressed individually (Table 5). Seven proteins were successfully expressed in *Bt* cells. The toxicity of Cry1Ab against *M. sexta* (*LC*_50_ 8.6 ng/cm^2^) was relatively low when compared to previous reports that have showed *LC*_50_ values two or threefold lower (Gómez et al., 2018; Pacheco et al., 2018). In contrast, the toxicity of this protein against *S. frugiperda* showed similar values to other reports (Gómez et al., 2018). However, it is important to mention that it was shown that different *S. frugiperda* populations showed great variability in their susceptibility to Cry1Ab, ranging from not susceptible at all, up to highly susceptible (Graser et al., 2017; Gómez et al., 2018; Figueiredo et al., 2019). The Cry1Bb protein showed to be highly active against *M. sexta* with *LC*_50_ value of 5.4 ng/cm^2^ and this protein was the most active toxin against *S. frugiperda* among all tested proteins in this work, with a *LC*_50_ value of 128.5 ng/cm^2^ (Table 5), showing two times higher toxicity to S. *frugiperda* than previously reported (Luo et al., 1999). The toxicity of Cry1Da against *M. sexta* and *S. frugiperda* was similar to previous reports (Höfte and Whiteley, 1989; Wang et al., 2019). In the case of Cry1Fb, Cry1Hb, and Cry1Ka there are no reports previous in the literature about the toxicity of these proteins against these target insects. It was only shown that Cry1Fb was active against *Agrotis ípsilon* and was not toxic against *Heliothis virescens* (de Maagd et al., 2003; Karlova et al., 2005); and that Cry1Ka was active against *Arfogeia rupae*, but not active against *Plutella xvlostella, Spodoptera exigua*, or *Bombyx mori* (Koo et al., 1995). Finally the toxicity of Cry1Ja was previously analyzed against *S. exigua* showing high toxicity (Herrero et al., 2001; Ibargutxi et al., 2006) and the reported toxicity of Cry1Ja against *M. sexta* was much higher than the *LC*_50_ value reported in this work (Herrero et al., 2001).

*Spodoptera frugiperda* has been shown to be highly susceptible to Cry1Fa and transgenic maize expressing Cry1Fa has been shown to be effective in controlling this insect pest. However, *S. frugiperda* has evolved resistance to Cry1Fa-maize in Puerto Rico, United States, Brazil, and Argentina (Storer et al., 2012; Farias et al., 2014; Chandrasena et al., 2018). Thus, additional Cry proteins that show no cross-resistance to Cry1Fa are likely to provide tools to counter resistance of this pest to transgenic maize. In the case of Cry1Bb, it was shown that this toxin and Cry1Fa share a binding site in the brush border membranes of *S. frugiperda* suggesting that Cry1Bb and Cry1Fa should show cross-resistance in this insect species (Luo et al., 1999). However, the Vip3Aa has been shown to be effective against the Cry1Fa resistant populations (Welch et al., 2015). The toxicity of the other PP proteins codified in *Bt* GR007 strain such as Cry1Id, Cry1Nb, Cry2Ad, the new Mpp, Vpa2Ac, Vpb1Ca Vip3Af, and Vip3Ag remains to be analyzed against different target insects including insect populations that have evolved resistance to other Cry proteins in the field.

Finally, we analyzed the promotor region of GR007 PP genes. We found that *cry1Da, cry1Ja, cry1Bb, cry1Hb, cry1Fb*, and *cry1Ka* genes have promotor regions highly similar to *cry1Ab* gene suggesting that all of them may be regulated by Sig^*E*^ and Sig^*K*^ factors. Previously the regulation of some *cry* genes from HD12 strain was analyzed showing that the expression of *cry1Ae, cry1Bb, cry1Fb*, and *cry1Ja* genes is regulated by both sigma factors, while *cry1Da* was only regulated by Sig^*E*^. However, the problem was that the upstream sequence of *cry1Da* gene was not fully analyzed (Song et al., 2017). They did not included the −35 region of Sig^*K*^ promoter, resulting in the loss of Sig^*K*^ function when fused to *LacZ* gene (Song et al., 2017). Here we show that the *cry1Da* gene that is present in GR007 strain has the complete promoter region including −35 and −10 DNA sequence for binding of both sigma factors, suggesting that its expression is determined by both sigma factors. Our analysis also showed that promotor regions from *cry1Nb, cry1Id*, and *cry2Ad* showed different sequences, suggesting that they do not share the same regulation as the other *cry* genes present in this strain (Supplementary Figure 3). Blast analysis showed that the promotor region from *cry2Ad* is similar to the upstream sequence of *cry2Ab* gene. The promotor region of *cry1Id* is similar to the upstream sequence of *cry1Ia*, while the promotor region of *cry1Nb* has no similarities to any other sequence including all known upstream sequences from other *cry* genes.

Overall, our data indicate that in nature certain *Bt* strains contain and express multiple PP genes codifying for proteins that display activities against different target pest. These strains could be excellent candidates to generate novel formulations. The information provided here regarding the genome of *Bt* GR007 strain will help with the understanding *Bt* toxin gene diversity and regulation of its PPs.

## Data Availability Statement

The data for this study can be found in BioProject: PRJNA736034 and Gene Bank Accession numbers CP076539, CP076541, CP076540, and CP076542.

## Author Contributions

JS collected the sample. SP, IG, and JS conducted the experiments. SP and MC analyzed the data. AB and MS conceived of the idea and designed the study. SP, MS, and AB participated in drafting the manuscript. All authors contributed to the article and approved the submitted version.

## Conflict of Interest

The authors declare that the research was conducted in the absence of any commercial or financial relationships that could be construed as a potential conflict of interest.

## Publisher’s Note

All claims expressed in this article are solely those of the authors and do not necessarily represent those of their affiliated organizations, or those of the publisher, the editors and the reviewers. Any product that may be evaluated in this article, or claim that may be made by its manufacturer, is not guaranteed or endorsed by the publisher.

## Footnotes

1
https://www.bpprc.org/

2
https://www.pacb.com/support/software-downloads/
