# Supplementary material for: Whole Genome Sequencing Analysis of Bacillus thuringiensis GR007 Reveals Multiple Pesticidal Protein Genes
Source: Front Microbiol. 2021 Nov 2;12:758314. doi: 10.3389/fmicb.2021.758314 (PMC8594373; doi:10.3389/fmicb.2021.758314)

Supplementary Material

1 Supplementary Data

Supplementary File S1. detailed information of *Bt* GR007 genome annotation.

Chromosome, pGR340, pGR157, pGR55 and Prophages

2 Supplementary Figures

Supplementary Figure S1. Circular plasmids maps. Left panel corresponds to comparison of plasmids pGR340 of *Bt* GR007 (brown outer circle) with pHD120345 of *Bt* HD12 (red inner circle). Right panel corresponds to comparison of plasmids pGR157 (orange outer circle) and pGR55 (brown middle circle) of *Bt* GR007 with pHD120161 of *Bt* HD12 (red inner circle). ORF for pesticidal proteins are indicated.

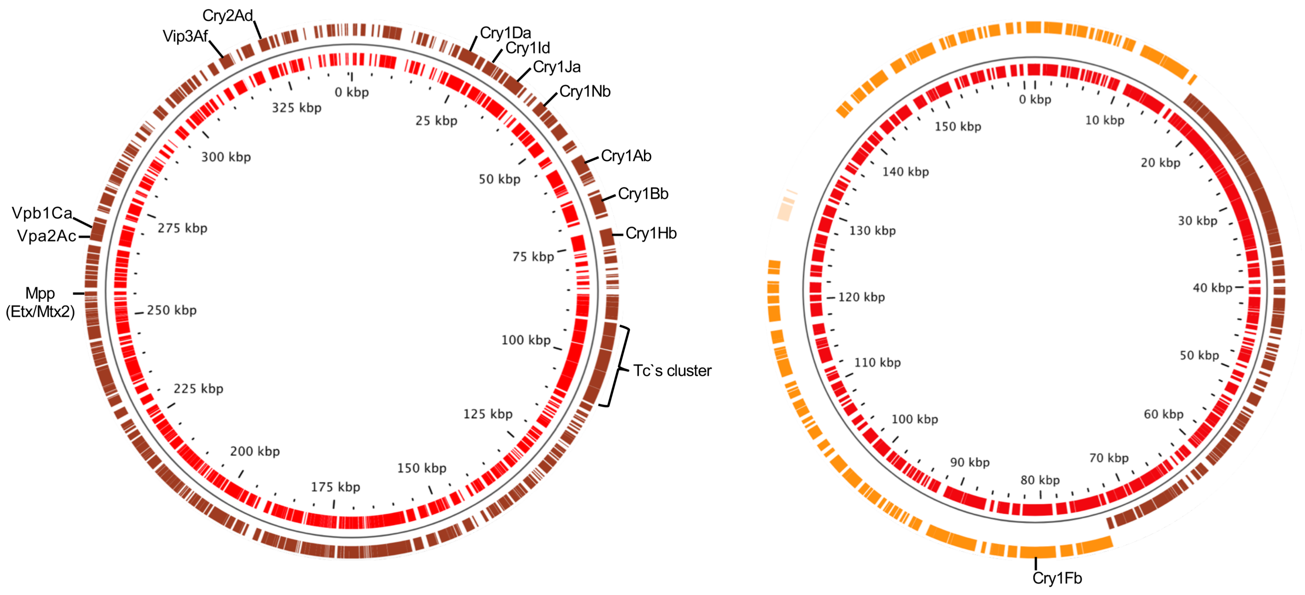

Supplementary Figure S2. SDS-PAGE analysis of the expression of Cry1Id and Cry2Ad proteins.

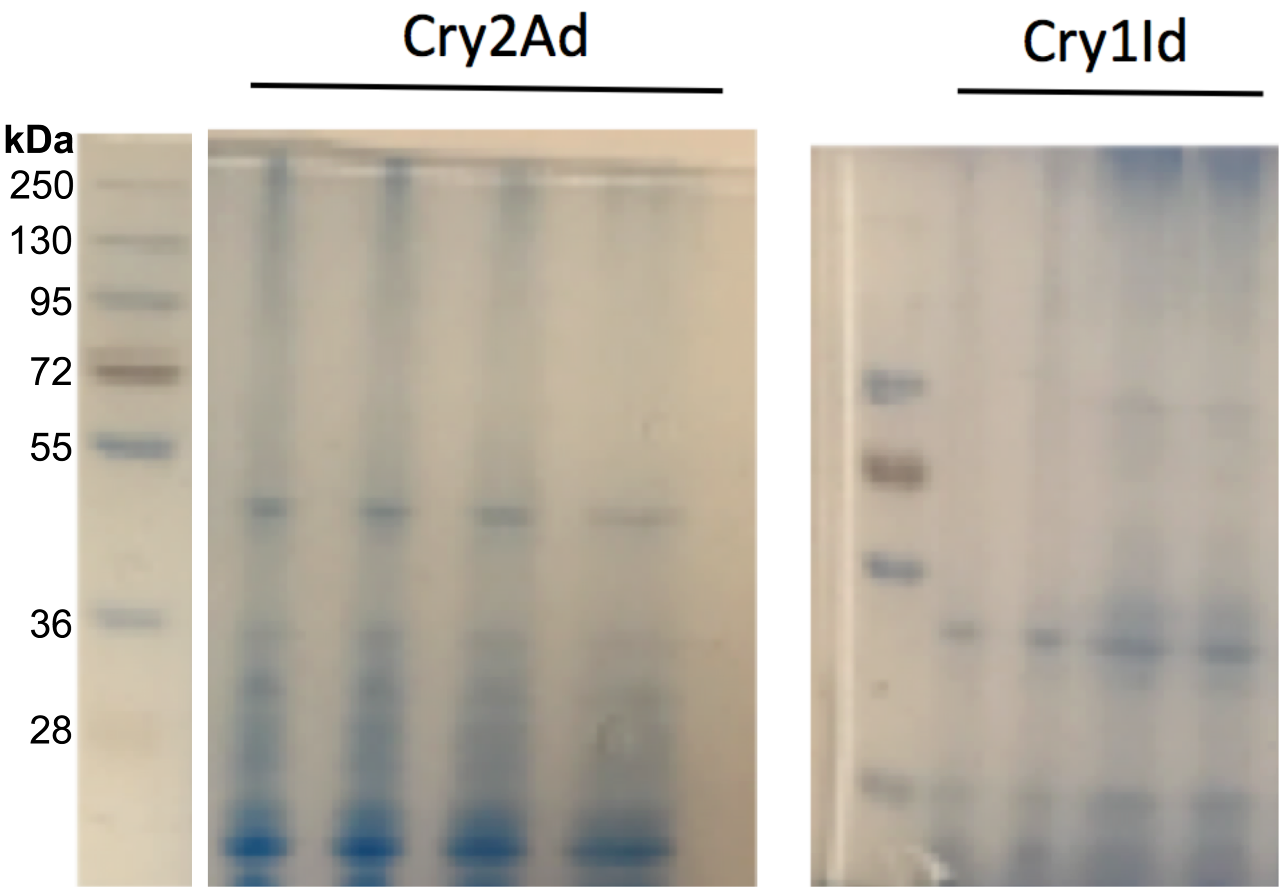

Supplementary Figure S3. Alignment of *cry* gene promotors. The putative -35 and -10 sequences for Sig^K and Sig^E factors are labeled with squares. Alignment was performed on T-coffee server using the option for align homologous promoter region (Pro-coffee).

.

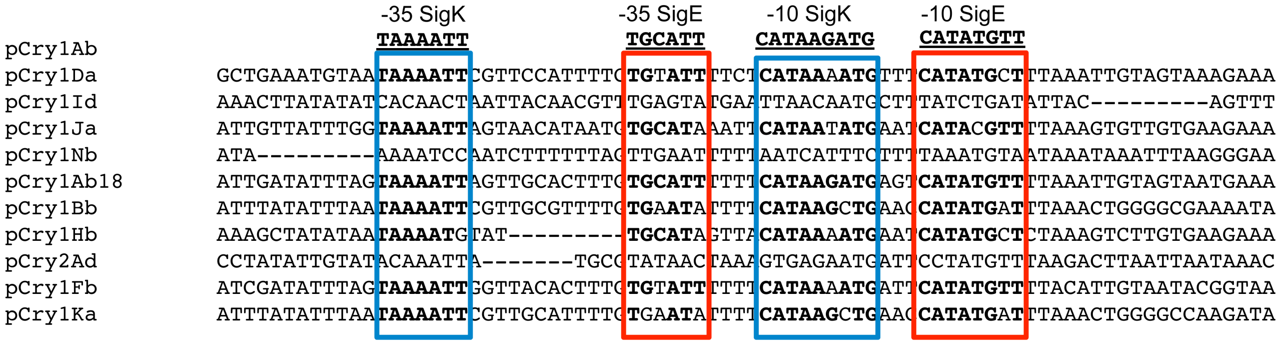

Supplement: Supplementary file 1 [file Data_Sheet_1.PDF]
